# Technical violations and infractions are drivers of disengagement from methadone treatment among people with opioid use disorder discharged from Connecticut jails 2014–2018

**DOI:** 10.1186/s13011-023-00541-2

**Published:** 2023-07-07

**Authors:** Phillip Marotta, Alissa Hass, Adam Viera, Molly Doernberg, Russell Barbour, Lauretta E. Grau, Robert Heimer

**Affiliations:** 1 Brown School, Washington University in St. Louis, 1 Brookings Dr., St. Louis, MO USA; 2 Indiana University School of Medicine, Bloomington, USA; 3 Yale School of Public Health, New Haven, USA; 4 Yale University, New Haven, USA

**Keywords:** Incarceration, Reentry, Methadone, Opioids, Criminal legal system

## Abstract

**Background:**

We investigated the interaction between arrests for technical violations vs. receiving new charges with receiving community-based methadone treatment on time-to reincarceration (TTR) in a cohort of men with opioid use disorder (OUD) released from custody from two Connecticut jails from 2014 to 2018.

**Methods:**

Hazard ratios (HR) were estimated for time to reincarceration for technical violations/infractions, misdemeanors only, felonies only, and both misdemeanors and felonies after adjusting for age, race/ethnicity, and receiving methadone treatment during incarceration or in the community following release. Moderation analyses tested the hypotheses that the benefits of receiving methadone in jail or the community on TTR were significantly different for people with only technical violations and infractions compared to misdemeanor and felony charges.

**Results:**

In the sample of 788 men who were reincarcerated, 29.4% received technical violations with no new charges (n = 232) with the remainder of the sample receiving new charges consisting of 26.9% new misdemeanor charges, 6.5% felony charges, and 37.2% both felony and misdemeanor charges. Compared to men who received new misdemeanor charges, TTR was significantly shorter among those who received technical violations and infractions with no new charges amounting to a 50% increase in TTR (334.5 days, SD = 321.3 vs. 228.1 days, SD = 308.0, p < 0.001; aHR = 1.5, 95% CI = 1.3, 1.8, p < 0.001). TTR of men who resumed methadone and were charged with a new crime was 50% longer than those who resumed methadone and received technical violations/infractions with no new charges. (230.2 days, SD = 340.2 vs. 402.3 days, SD = 231.3; aHR = 1.5, 95%CI = 1.0, 2.2, p = 0.038).

**Conclusions:**

Reducing technical violations may enhance the benefits of providing community-based methadone following release from incarceration on extending the time between incarcerations during the vulnerable time post-incarceration and reduce the burden on correctional systems.

## Introduction

The prevalence of opioid use disorders (OUD) among people who are incarcerated in the United States (US) continues to rise, emphasizing the responsibility of the carceral health care system to deliver effective evidence-based treatments for OUD [1–5]. The overwhelming majority of people with OUD who are incarcerated do not receive medication treatment for opioid use disorders (MOUD) that include methadone, buprenorphine and naltrexone and as a result suffer involuntary withdrawal, often without adequate medical and behavioral support [6, 7]. To address these concerns, some jurisdictions have offered MOUD in their correctional systems. Post-release outcomes of overdose, continuation of methadone and buprenorphine upon release, ongoing drug use, and recidivism have been evaluated in some jurisdictions offering MOUD in carceral settings [8–17] and in subsequent systematic reviews [18–20]. Results have consistently supported the effectiveness of methadone treatment for OUD as a gold-standard strategy in preventing overdose and continuing treatment after transitioning into the community from periods of incarceration [5, 15–17, 21, 16]. Data from Rhode Island and New York City suggest providing buprenorphine in carceral settings can reduce overdose mortality [22–24]. Limited attention has been paid to investigate benefits of receiving methadone and buprenorphine treatment while incarcerated on subsequent reincarceration and results among published reports are contradictory. Studies from Connecticut [7] and Baltimore [9] in the US, France, New South Wales in Australia, and Canada found no difference in the proportion of individuals reincarcerated while a study from Albuquerque (US) reported a delay in reincarceration if individuals received MOUD while incarcerated [10, 13, 15–17]. Studies from Connecticut, Australia, and Canada found lower risk of reincarceration among individuals who resumed methadone or buprenorphine following release from prison and jails [14, 15].

Many of those released from custody are placed on some form of community supervision consisting of probation and parole [25–27]. One quarter of the 3.8 million people who are on probation or parole in the US were incarcerated from drug-related offenses [26]. Recent research has called attention to the disproportionate representation of people with OUD within populations of people who are incarcerated, many of whom experience a revolving door of incarceration [28–31]. Supervision violations of probation or parole account for 45% of new prison admissions in the US of which 25% are due to technical violations [32].

Prior research has found that intensity of supervision is associated with increased risk of violations and reincarceration which are the greatest for absconding [33, 34]. Research suggests that violation of supervision does not attenuate the risk of engaging in future recidivism and in certain circumstances can increase risk. Studies have found that technical violations account for up to 80% of returns to incarceration with new charges accounting for a relatively small proportion costing up to 2.8 billion dollars [35, 36]. Violations can include failure to appear at appointments with probation officers and court appearances, possession of illicit drugs or their presence in toxicology screens, motor vehicle infractions, outstanding warrants, non-payment of civil fines, and refusal to follow up with referrals to mandated substance use treatment or social services [37, 29]. People who do not resume methadone/buprenorphine treatment in the community may be at greater risk of receiving technical violations and infractions because of having mandates added as conditions of their release including follow-up with treatment.

A recent study of people with OUD who were recently released from incarceration in Massachusetts investigated risk of recidivism and found that receiving buprenorphine in jail reduced arraignments and reincarceration but not violations of probation 31. It did not examine how continuing treatment in the community shaped reductions in recidivism. This study did not provide information on the pathway of generating arrest warrants out of probation violations and the process of deciding arraignments. Greater research is needed that teases apart the impact of engagement in the community on the pathway of technical violations, arrest warrants from probation violations and the process of deciding arraignments. This missing element is particularly important because engagement in treatment as a condition of release is strongly linked to technical violations. community. Being on probation and parole can increase risk of experiencing technical and violations of probation that involve appearing in court, treatment, or other dimensions of substance use disorder treatment.

The unified corrections system in Connecticut, wherein a single state agency oversees all jails and prisons, provides a unique opportunity for such an examination. We recently reported findings from a retrospective case-control study of a pilot program that offered methadone to men incarcerated in two jails in Connecticut [8]. Treatment was offered only to those who had been receiving methadone at the time of their incarceration and was dependent on the treatment capacity within the jails. As a result, only 42% of eligible men continued treatment throughout their time in jail. Our study revealed that following release from custody, treatment while in jail resulted in a lower rate of overdose and greater resumption of methadone treatment, consistent with other studies exploring these outcomes. Although there were no overall differences in reincarceration following release from custody, resumption of methadone following release from custody was associated with a significant time delay to reincarceration [8]. One issue unaddressed in the prior publication was a determination whether the reasons for reincarceration differed between those who received methadone and those who did not receive methadone treatment, either while incarcerated or following release from custody. Investigating differential pathways to reincarceration and the impact of engaging in MOUD treatment following initial release from custody could identify potential avenues of intervention to increase engagement in care and reduce reincarceration.

Recently incarcerated persons who are on probation and parole may be at greater risk of experiencing technical violations and violations of probation that consist of not following up with treatment or other aspects of substance use disorder treatment, court appearances, positive urine toxicology screens and other forms of not following up with mandated conditions of release. People diagnosed with OUD may experience more punitive responses from probation and parole officers that result in violations of probation and technical violation without any additional charges. In these instances, reincarceration occurs without going through adjudication for a new case. Technical violations of probation or parole are not by themselves criminal offenses (e.g., failing to report for a scheduled office visit, missing a curfew, lack of employment or attendance at school, testing positive for drug or alcohol use, failure to appear and some driving offenses). People with technical violations may experience rearrest more quickly if they return to drug use in the community. Studies are yet to examine if possible, benefits of MOUD on reincarceration are differentially experienced by people following release from custody who are at risk of technical violations compared to those who commit misdemeanors and felonies. Technical violations offer opportunities for enhanced treatment, linkage to services and policies that divert people into treatment rather than incarceration.

### The present study

The State of CT initiated a pilot program to provide methadone to individuals in two mens’ jails to individuals who, prior to being jailed. Individually identified data were obtained from Connecticut Department of Correction (CT DOC) for 1,564 men eligible for treatment who had been jailed between October 2013 and December 2018. DOC provided the research team dates of release and reincarceration, provision of methadone treatment in jail, and demographics. Only 660 (42.2%) of those eligible received treatment throughout their time in custody because the pilot program had limited treatment capacity. The first published paper using this dataset found that receiving methadone while in jail was not associated with a decrease in the percent of men reincarcerated nor in the TTR. However, we did not look at whether these effects varied as a function of the reason for reincarceration. The focus of the present manuscript is to test the hypothesis that time until reincarceration co-varied by characteristics of the reason for reincarceration. The sample was restricted to include a subset of 788 men because they experienced reincarceration during the study period from 2014 to 2018. The present study added data from court records on the charges that led to the reincarceration of 788 of the 1564 men to the compiled data from the initial study [7].

## Methods

### Data

For this study, the data on the 788 men who were reincarcerated were linked by the CT Department of Mental Health and Addiction Services (DMHAS) to data concerning resumption of methadone following release. Individual cases (those treated throughout their initial time in jail) and controls (those either not started on methadone or whose treatment was terminated) were matched to DMHAS records to identify individuals resuming methadone and the date of resumption. Because all men in the study were being treated with methadone prior to their initial incarceration, post-release treatment is referred to as ‘resumption of community methadone.’

For this study, linked data on reasons for reincarceration were supplied from the CT Judicial Branch Court Special Services Division. Offenses leading to reincarceration were analyzed based on the CT General Statute and collapsed into categories.

***Severity of charges***. A hierarchical categorical variable was created in ascending order indicating if all the offenses leading to reincarceration were (1) only technical violations and infractions with no new criminal charges, (2) only new misdemeanor charges, and (3) only new felony charges. We also created a composite variable that included both new felony and misdemeanor charges. In the hierarchy, individuals with charges in multiple categories were assigned to the more severe. In addition, the CT-Uniform Crime Reporting (UCR) codes enumerated in the Connecticut General Statutes were used to create variables for each of the crime types: (1) drug crimes, (2) property crimes, (3) offenses against public peace and safety, (4) obstruction, (5) violent crimes as well as a category for miscellaneous other crimes.

**Time-to-reincarceration (TTR)** was calculated using data from the CT DOC as the number of days between release from and reincarceration in the state correctional system.

***Receiving methadone treatment during incarceration and in the community*** was constructed as a pair of dichotomous variables measuring whether methadone was received throughout incarceration and/or resumed in the community following release from custody, creating four mutually exclusive categories.

***Race and ethnicity*** were reported by the CT-DOC as (1) Black, (2) White, (3) Hispanic, and (4) other, including Asian, Native American, or multi-racial in the CT DOC dataset. **Age** was categorized into 18–23, 24–29, 30–39, 40–49, and > 50 years.

### Statistical analyses

Descriptive analyses included summary statistics of reasons for reincarceration and demographic characteristics. Crime types were examined only for the descriptive analyses. Bivariate tests of significant differences between resumption of methadone in the community and all the independent variables were performed using Mann-Whitney (2 groups) and Kruskal-Wallis (2 or more groups) tests for categorical variables because of their capacity to identify group differences and handle non-normally distributed continuous data [38–42]. TTR was stratified by severity of charges, reasons for reincarceration, and resumption of methadone in the community. Survival curves of the hazard rates of TTR by severity of charges and resumption of treatment were visualized to evaluate the data for the potential of interaction effects [43]. Diagnostic analyses were performed to estimate associations between a categorical variable with categories of (1) treatment while in jail only, (2) treatment in the community only, (3) treatment in jail and in the community, with (4) no treatment as the referent group. To increase the efficiency and parsimony of the model we only included resumption of community methadone in the main and interaction effects models.

In our preliminary analyses, crime type was not significantly associated with a difference in TTR and none of the interactions were significant and as a result was only provided in the descriptive results section. Survival regression analyses were performed on charge types and their interactions with resumption of methadone and were not found to be significantly associated with TTR. The TTR did not significantly differ by the type of charges. These insignificant results are not included in this analysis as they were not part of our core hypothesis and question. Instead, descriptive data for crime type was included in descriptive statistics.

Unadjusted and adjusted time-to-event analyses modeled the impact of severity of charges, resumption of methadone in the community, treatment in jail, and demographic characteristics on TTR [43]. We performed several diagnostic analyses prior to arriving at our final regression models. The first step determining that proportional hazards assumptions using the log rank test with equality of survivor functions were violated [44, 45]. Thus, survival curves were parameterized using semi-parametric accelerated failure time models [44–46]. A Weibull model, which allows a continuous probability distribution that is flexible to fit an extensive range of data distributions [47, 48], was created with variables of resumption of community methadone and severity of charges to estimate the effects of severity of charges – technical violations/infractions, misdemeanors only, felonies only, and misdemeanors and felonies – and resumption of treatment on TTR after adjusting for demographic covariates [44–46]. Hazard ratios (HR) were further adjusted for age, race/ethnicity, receiving treatment during incarceration, and resumption of community treatment.

Moderation analyses were performed to test if the effects of resuming community treatment on TTR varied as a function of the severity of charges and reasons for reincarceration. The next step of the regression models consisted of including interaction terms of resumption of treatment by severity of charges with receiving methadone + misdemeanor only as the referent category. All statistical analyses were performed using STATA v16 [49].

## Results

### Descriptive statistics

Descriptive statistics for the subsample of 788 men who were reincarcerated are provided in Table 1. Significant tests results for the Mann-Whitney and Kruskal Wallis are shown using p-values testing for significant differences between days to reincarceration and resumption of methadone for severity of charges and demographic characteristics, and the interaction between resumption of methadone and severity of charges. The initial analysis of the raw remand to custody data created charge-level categories measuring severity of charges as well as types of offenses. There was a total of 3,407 charges across the whole sample of 788 men who were incarcerated, amounting to an average of 4.1 (SD = 4.3) new charges per person. **Severity of charges.** Technical violations and infractions with no new charges led to incarceration of 29.4% (n = 232) of the sample. Participants reincarcerated for new charges involving misdemeanors with no felonies accounted for 26.9% (n = 212), felonies with no misdemeanor charges accounted for 6.5%, (n = 51), and the combination of new felony and misdemeanor charges accounted for 37.2% (n = 293) of readmissions to incarceration. **Types of new charges leading to reincarceration**. Property offenses were the most common new charges, involved in 40.5% of all new reincarcerations, followed by flight or failure to appear, disturbance of public peace, drug law offenses, part 1 crimes, obstruction of justice, forgery/fraud, and all other offenses. Men who received methadone in jail accounted for 43.3% (n = 341) of those reincarcerated while, of those reincarcerated, 58.5% (n = 461) had resumed community treatment.

**Table 1:** Charges Leading to Reincarceration. Frequencies and proportions of severity of charges, types of crimes and demographic characteristics of 788 study participant who experienced reincarceration

	Overall
	%(n)
Severity of charges	
Misdemeanor - no felony charges	26.9 (212)
Violation/infraction only - No criminal charges	29.4 (232)
Felony - no misdemeanor charges	6.5 (51)
Combination of felony and misdemeanor charges	37.2 (293)
Property	40.5 (319)
Violent Offense	37.2 (293)
Flight/failure to appear	24.8 (195)
Part 1 crimes	21.2 (167)
Public Peace	23.1 (182)
Obstruction	12.8 (101)
Forgery/Fraud	15.7 (124)
Misc/other/weapons/violation order of protection	12.2 (96)
Type of drug crime	
None	77.7 (612)
Possession only	15.5 (122)
Trafficking only	5.5 (43)
Trafficking and possession	1.4 (11)
Location	
New Haven	60.2 (474)
Bridgeport	39.9 (314)
Race/Ethnicity	
White	62.1 (489)
Black	12.2 (96)
Hispanic	21.6 (170)
Other	4.2 (33)
Age (years)	
23–29	7.9 (62)
30–39	44.0 (347)
40–49	26.8 (211)
50+	21.3 (168)
Treated in jail	43.3 (341)
Resume Methadone in the Community	58.5 (461)

Part 1 crimes consist of Murder, Assault, Rape and Arson

### Main effects of severity of charges and resumption of treatment on TTR after adjusting for sociodemographic characteristics

Table 2 presents mean TTR by severity of charges, treatment during incarceration, and resumption of community methadone, the model’s unadjusted (uHR) and adjusted hazard rates (aHR) and p-values for statistical tests of significance. Methadone resumption in the community significantly increased TTR by 47.1% compared to men who did not (SD = 320.5, p < 0.001 vs. 217.9 days, SD = 287.9; 345.4 days; aHR = 0.6, 95% CI = 0.5, 0.7, p < 0.001; uHR = 0.6, 95% CI = 0.5, 0.7, p < 0.001). Compared to men who received new misdemeanor charges, TTR was significantly shorter among men who received technical violations or infractions with no new charges (334.5 days, SD = 321.3 vs. 228.1 days, SD = 308.0, p < 0.001; aHR = 1.5, 95% CI 1.3, 1.8, p < 0.001; uHR = 1.6, 95% CI = 1.3, 1.9, p < 0.001), amounting to a 50% shorter TTR (Table 2). No significant difference was identified in TTR between those who were reincarcerated due to felonies and misdemeanors compared to misdemeanors only. In the unadjusted models, TTR was not different for men jailed in New Haven versus Bridgeport (313.0 days SD = 347.5 vs. 261.7, SD = 351.4, p = 0.081) but the HR was lower for the men jailed in New Haven (uHR = 0.9, 95%=0.7, 0.9, p = 0.030).

**Table 2:** Unadjusted and adjusted hazard ratios estimating main effects between severity of charges, resumption of treatment and TTR (n = 788)

	Mann-Whitney/Kruskal-Wallis tests					
	Days (SD)	p-value	uHR	p-value	aHR	p-value
Severity of charges		**< 0.001**				
Misdemeanor - no felony charges	334.5 (321.3)		Ref		Ref	
Violation/infraction only - no criminal charges	228.1 (321.2)		**1.6 (1.3, 1.9)**	**< 0.001**	**1.5 (1.3, 1.8)**	**< 0.001**
Felony - no misdemeanor charges	297.2 (308.0)		1.1 (0.8, 1.5)	0.435	1.1 (1.8, 1.5)	0.476
Combination of felony and misdemeanor charges	312.5 (296.1)		1.1 (0.9, 1.3)	0.431	1.1 (0.9, 1.3)	0.451
Resume Methadone in the Community		**< 0.001**				
Yes	292.5 (313.6)		**0.6 (0.5, 0.7)**	**< 0.001**	**0.6 (0.5, 0.7)**	**< 0.001**
No	217.9 (287.9)		Ref		Ref	
Treated in jail		0.256				
Yes	308.8 (312.8)					
No	280.1 (314.1)					
Location		**0.081**				
New Haven	313.0 (347.5)		**0.9 (0.7, 0.9)**	**0.030**	0.9 (0.8, 1.0)	0.103
Bridgeport	261.7 (351.4)		Ref		Ref	
Race/Ethnicity		**0.043**				
White	297.2 (302.0)		Ref		Ref	
Black	330.3 (439.1)		1.0 (0.8, 1.3)	0.874	0.9 (0.7, 1.2)	0.427
Hispanic	276.0 (266.7)		1.1 (0.9, 1.3)	0.392	1.0 (0.9, 1.2)	0.721
Other	199.9 (254.9)		**1.5 (1.1, 2.1)**	**0.026**	1.3 (0.9, 1.9)	0.170
Age (years)		0.31				
23–29	353.7 (363.2)		Ref		Ref	
30–39	273.0 (282.8)		1.3 (0.9, 1.7)	0.056	1.3 (1.0, 1.7)	0.102
40–49	286.6 (276.3)		1.2 (0.9, 1.7)	0.136	1.2 (0.9, 1.5)	0.336
50+	317.9 (388.2)		1.2 (0.9, 1.6)	0.221	1.1 (0.8, 1.5)	0.422

uHR: unadjusted Hazard Ratio; aHR adjusted Hazard Ratio; SD: Standard Deviation; * p < 0.05; **p < 0.01; ***p < 0.001; ref: Referent category for categorical variable in regression; Models with interaction terms adjusted for age, race, and location

### Assessment of interaction between severity of charges and resumption of methadone on TTR

**Fig. 1:**
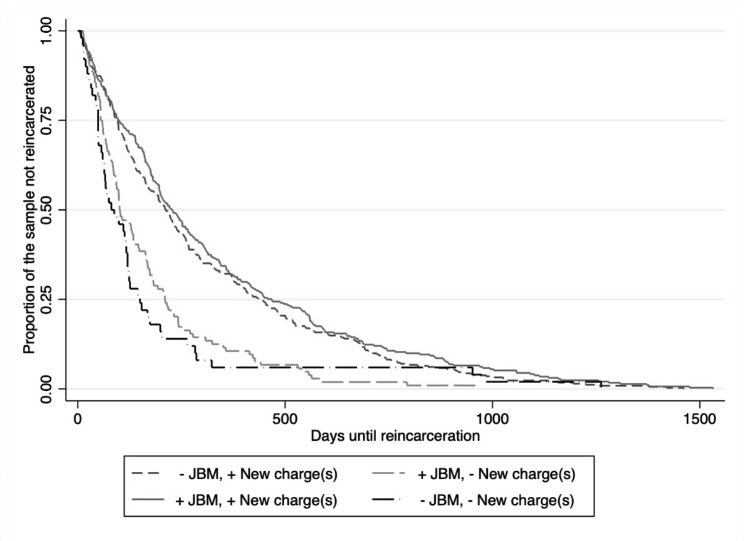
Survival curves of TTR by severity of charges and receiving jail-based methadone (JBM)

**Fig. 2:**
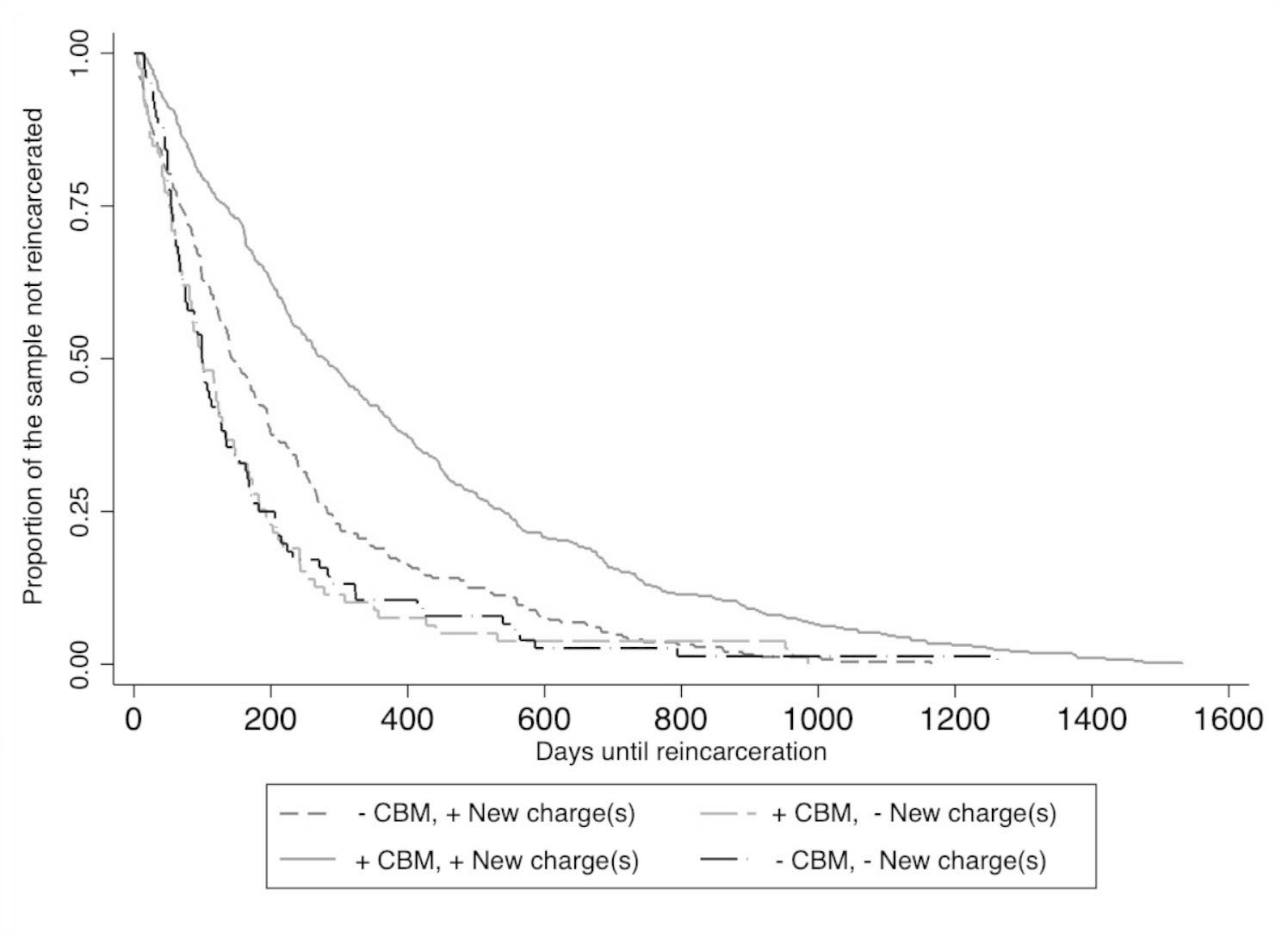
Survival curves of TTR by severity of charges and resumption of community-based methadone (CBM)

Figure 1 presents survival curves of TTR by severity of charges and receipt of jail-based methadone. Figure 2 presents survival curves of TTR by severity of charges (any new criminal charge versus technical violation) and resumption of community-based methadone. The survival curves for TTR were nearly identical for men with technical violations/infractions regardless of resumption of methadone and for men with new criminal charges who resumed methadone (Figs. 1 and 2).

***Statistical assessment of resumption of methadone by severity of charge interaction effects.*** The data in Table 3 presents the interactions between receiving methadone in the community and whether reincarceration resulted from technical violations/infractions with no new charges or new charges consisting of misdemeanor and felony offenses without violations or infractions. The mean number of days to reincarceration was stratified by resumption of community methadone to illustrate differences in the TTR by severity of charges. On average, TTR for men with technical violations/infractions who received methadone in the community was only four days longer than their counterparts who did not receive treatment (230.2 days SD = 340.2 vs. 225.7, SD = 397.5). The difference in mean survival times of men who resumed community methadone after release was ≥ 177.47 days for misdemeanor with no felony charges, ≥ 201.22 days for felony with no misdemeanor charges, and ≥ 165.88 days for combination of felony and misdemeanor charges among those treated compared to those who were not treated. TTR of men who resumed methadone and received technical violations/infractions with no new charges was significantly shorter than men who resumed methadone and were charged new crime (230.2 days, SD = 340.2 vs. 402.3 days SD = 231.3, p < 0.001) amounting to a 50% increase in TTR (aHR = 1.5, 95% CI = 1.0, 2.2, p = 0.038). None of the other interaction terms were significantly associated with TTR after adjusting for potential confounders. No significant differences were observed for TTR by jail-based treatment (results available upon request).

**Table 3:** Categorical by dichotomous interactions between receiving methadone and dummy variables of charge level with misdemeanor as the reference group (n = 788)

	TTR Mann-Whitney/Kruskal-Wallis tests							
	+Resume	−Resume	Mean Diff.					
	Days (SD)	Days (SD)	Days	p-value	uHR	p-value	aHR	p-value
Resume methadone							**0.6 (0.4, 0.7)**	**< 0.001**
Misdemeanor - no felony							ref	
Violation/infraction only							1.2 (0.9, 1.6)	0.184
Felony - no misdemeanor							1.2 (0.8, 1.9)	0.413
Felony and misdemeanor							1.1 (0.8, 1.5)	0.604
Resume*Charge level				< 0.001				
Resume*Misdemeanor	402.3 (349.8)	224.8 (231.3)	-177.47		ref	ref.		
Resume*Violation/Infr.	230.2 (340.2)	225.7 (397.5)	-4.5		**1.5 (1.1, 2.2)**	**0.028**	**1.5 (1.0, 2.2)**	**0.038**
Resume*Felony	399.8 (363.5)	198.6 (207.3)	-201.22		0.9 (0.5, 1.6)	0.685	0.9 (0.5, 1.6)	0.604
Resume*Misd/Felony	375.9 (324.6)	210.1 (206.7)	-165.88		1.0 (0.7, 1.4)	0.996	1.0 (0.7, 1.4)	0.951
**Model 1**								
**Community treatment status**								
Resume methadone							**0.6 (0.4, 0.7)**	**< 0.001**
**Charges**								
Misdemeanor - no felony							ref	
Violation/infraction only							1.2 (0.9, 1.6)	0.184
Felony - no misdemeanor							1.2 (0.8, 1.9)	0.413
Felony and misdemeanor							1.1 (0.8, 1.5)	0.604
**Model 2: Interaction effects**								
Community treatment status*Charges				< 0.001				
Resume*Misdemeanor	402.3 (349.8)	224.8 (231.3)	-177.47		ref	ref.		
Resume*Violation/Infr.	230.2 (340.2)	225.7 (397.5)	-4.5		1.5 (1.1, 2.2)	0.028	**1.5 (1.0, 2.2)**	**0.038**
Resume*Felony	399.8 (363.5)	198.6 (207.3)	-201.22		0.9 (0.5, 1.6)	0.685	0.9 (0.5, 1.6)	0.604
Resume*Misd/Felony	375.9 (324.6)	210.1 (206.7)	-165.88		1.0 (0.7, 1.4)	0.996	1.0 (0.7, 1.4)	0.951

uHR: unadjusted Hazard Ratio; aHR adjusted Hazard Ratio; SD: Standard Deviation; * p < 0.05; **p < 0.01; ***p < 0.001; ref: Referent category for categorical variable in regression; Models with interaction terms adjusted for age, race, and location

## Discussion

We analyzed merged databases from three state agencies in Connecticut to better understand factors that influence the likelihood of reincarceration in a cohort of men with OUD who had been receiving treatment with methadone at the time they were jailed. We focused on the impact of resuming methadone treatment following release from jail and on the charges that led to their reincarceration. Findings from our study suggest that TTR was shorter for men who received technical violations and infractions compared to any new charges. Findings from our interaction analyses suggest technical violations may reduce the beneficial impacts of resuming community methadone, in terms of delaying TTR compared to men who were charged with new crimes [5]. Findings from our study show as much as a 200-day increase in TTR except in the case of technical violations. These findings do not undermine the importance of providing community-based methadone treatment following release but rather underscore the need to provide supportive alternatives to reincarceration based on technical violations to ensure that people with OUD stay in the community.

### Limitations and avenues of future research

There are several limitations worth noting and each might give rise to fruitful avenues of future research. Our study dataset included a limited number of sociodemographic variables and did not measure whether participants were receiving other interventions alongside methadone treatment. We did not examine or consider crime types (i.e., property versus drug sales versus violent offenses) but rather compared effects across broad categories of charges. This paper did not examine whether the revocation process is faster than that for a new charge. The data necessary to assess whether the revocation process was not available for this study. It was not possible to determine if multiple offenses occurring on the same day would a revocation result in a faster return to jail. Additional research is needed to determine if the times of multiple offenses, the speed with which probation officers report violations to court officials and other nuanced factors are associated with increased speed of returning to jail.

This study generalizes only to jail and community-based corrections and not to prisons. Additionally, the study was conducted at only 2 sites and since this study methadone has expanded to 10 of the 13 jails and prisons in the state. Future research must examine how other medications including buprenorphine and Vivitrol impact recidivism using similar designs than prior studies with overdose as the outcomes. Prior studies suggest that buprenorphine and Vivitrol may have similar impacts. Future research must investigate if there is something specifically unique about methadone or if similar results are seen across the other types of medications.

While complicated to achieve, future research must create discrete categorical variables to elucidate the impact of methadone (or any other attempt to keep people with OUD out of the corrections system) on reducing specific types of crimes and the consequent incarcerations. This study only examined the effects of jail- and community-based incarceration on reincarceration for people who were receiving methadone prior to incarceration. Greater research is warranted that elucidates the effects of initiating methadone treatment in correctional settings for people who were not previously receiving treatment. Future research must investigate the impact of resuming methadone on employment, educational, family stability, and psychosocial needs through improved functioning, all of which might reduce recidivism.

Additionally, this study did not include types or frequencies of the activities underlying the technical violations. We compared charges resulting from technical violations only to charges that included new misdemeanor or felony charges with or without violations. We did not investigate whether the types or frequencies of activities underlying the technical violations could lead to a faster time until reincarceration. This would create many additional categories of comparison and were outside of the of the research hypothesis and the statistical power of sample to assess. This is nonetheless an important line of future inquiry.

Health and well-being are negatively impacted by reincarceration. Reincarceration cuts important social ties, relationships with the health care and social service system, and peer networks resulting in greater risk of homelessness and overdose upon return to the community. Our prior report found that methadone treatment after release from custody attenuated the likelihood of reincarceration [5]. The current study deepens the existing body of literature by examining whether the benefit of receiving methadone varied across different reasons for reincarceration and points to a major reason for the revolving door of incarceration among people with OUD. The most troubling part of this finding is that treatment with methadone increase TTR except for those brought to court because of technical violation alone. (Figures 1 and 2). Findings from this study suggest that technical violations of probation may diminish the positive public health effects of receiving community methadone following incarceration and shorten the time spent in the community free from reincarceration.

### Implications for practice and policy

The implications of our investigation provide evidence that can be considered within jurisdictions that are identifying critical points of intervention to reduce the funneling of people with substance use disorders into the criminal justice pipeline. Our findings suggest that technical violations without any new charges is a major point of reentry into the carceral system and out of the community-based addiction treatment system. Policymakers and legislators must consider technical violations as a critical point to promote intervention to reduce incarceration. Such interventions could enhance resources to adhere to the strictures of probation, deliver training to prevent the re-incarceration of people with OUD. The Sequential Intercept Model provides a useful policy framework for integrating greater diversionary resources into post-jail services that prevent reincarceration through technical violations [50, 51]. Our findings support current initiatives to expand the use of SIM as an approach to deploying social and health care resources to critical points of recidivism along the criminal legal pipeline.

Implications of our findings support successful approaches to train probation officers to provide alternatives to issuing technical violations, including providing greater social and health care resources to people with OUD. Potential trainings could include motivational interviewing and/or linkage to care models that involve social workers, peer navigators or community health workers. Research is needed that focuses on whether bolstering social and health care resources as an alternative to incarceration will reduce the frequency of technical violations.

It should not be inferred as an implication from this study that methadone treatment is the cause of technical violations, higher likelihood of reincarceration and therefore questioning its use for this population. Instead, departments of corrections in state and local jurisdictions, criminal legal practitioners and clinicians can use this information to justify research into alternatives to reincarceration to deploy at the time of a possible technical violation to increase support in the community as opposed to resorting to rearrest and reincarceration. A clear implication of this study is the issuance of technical violations to people with OUD diminished the impact of methadone on reincarceration-free time in the community. A conclusion from this analysis is that technical violations are what is needed to be changed in the criminal legal system and replaced with enhanced supportive interventions that increase access to familial supports, employment/vocational and educational options in the community. Findings from this study lend support for enhancing community-driven efforts to get people with prior criminal justice involvement engaged in care following release from carceral settings. Focusing greater social and health care resources on diversion away from criminal legal system involvement could prevent and reduce reincarceration.

## Data Availability

Data is available through a request process upon request.
